# Cognitive Test Scores in UK Biobank: Data Reduction in 480,416 Participants and Longitudinal Stability in 20,346 Participants

**DOI:** 10.1371/journal.pone.0154222

**Published:** 2016-04-25

**Authors:** Donald M. Lyall, Breda Cullen, Mike Allerhand, Daniel J. Smith, Daniel Mackay, Jonathan Evans, Jana Anderson, Chloe Fawns-Ritchie, Andrew M. McIntosh, Ian J. Deary, Jill P. Pell

**Affiliations:** 1 Institute of Health & Wellbeing, University of Glasgow, Glasgow, Scotland, United Kingdom; 2 Centre for Cognitive Ageing and Cognitive Epidemiology, Department of Psychology, University of Edinburgh, Edinburgh, United Kingdom; Texas Tech University Health Science Centers, UNITED STATES

## Abstract

UK Biobank includes 502,649 middle- and older-aged adults from the general population who have undergone detailed phenotypic assessment. The majority of participants completed tests of cognitive functioning, and on average four years later a sub-group of N = 20,346 participants repeated most of the assessment. These measures will be used in a range of future studies of health outcomes in this cohort. The format and content of the cognitive tasks were partly novel. The aim of the present study was to validate and characterize the cognitive data: to describe the inter-correlational structure of the cognitive variables at baseline assessment, and the degree of stability in scores across longitudinal assessment. Baseline cognitive data were used to examine the inter-correlational/factor-structure, using principal components analysis (PCA). We also assessed the degree of stability in cognitive scores in the subsample of participants with repeat data. The different tests of cognitive ability showed significant raw inter-correlations in the expected directions. PCA suggested a one-factor solution (eigenvalue = 1.60), which accounted for around 40% of the variance. Scores showed varying levels of stability across time-points (intraclass correlation range = 0.16 to 0.65). UK Biobank cognitive data has the potential to be a significant resource for researchers looking to investigate predictors and modifiers of cognitive abilities and associated health outcomes in the general population.

## Introduction

### Background

Cognitive ability is important in epidemiological research, as a risk factor for health-related outcomes and as an outcome in its own right. Lower mental ability is associated with increased cardiovascular disease incidence [1] and earlier mortality [2], and cognitive decline is among the most feared aspects of ageing [3]. The global prevalence of dementia is expected to increase to 65.7 million people by 2030 [4], and it is therefore important to understand the predictors and modifiers of cognitive ability (and decline) into older age. It is essential that cognitive assessments used in epidemiological studies are valid, sensitive to underlying mental abilities and sufficiently reliable.

UK Biobank is a large general population cohort of more than 500,000 middle and older-age adults who underwent medical, sociodemographic, mental health and cognitive assessment between 2006 and 2010 [5,6]. Participants were recruited from a range of backgrounds and demographics. The cohort will be followed up at intervals, and morbidity and mortality tracked through linkage with routine medical records [5,6].

The baseline cognitive assessment in UK Biobank included brief bespoke tasks delivered in a novel computerised format. Around 20,000 participants subsequently underwent repeat assessment, including most of the cognitive battery [5]. It is important to establish that these novel cognitive tests are reliable if they are to be used to detect cognitive change on long-term follow-up.

One of the most replicated phenomena in psychology is that cognitive scores inter-correlate[7], possibly because their brain substrates have been subject to shared genetic/environmental influences (the *‘Common cause hypothesis’* [8]). This means that performance on one task (e.g. visuospatial memory) would be expected to correlate significantly with performance on another (e.g. information processing speed). This is commonly referred to as a general factor of cognitive ability (simply ‘*g*’), and is often investigated with principal components analysis (PCA), which identifies how much variance is shared between the tests[9]. It is important to quantify a *g* factor score based on the cognitive tasks administered in UK Biobank; not least because it is a common phenotype for genetic studies [10].

We aim to firstly test for and then describe aspects of a *g* factor in UK Biobank with PCA using baseline cognitive data. We also quantify the stability of cognitive scores in the participants using the repeat cognitive data. We intend for this to provide a useful resource and basis for future studies using cognitive data in UK Biobank.

## Method

### Participants

The UK Biobank cohort recruited 502,649 participants, aged 40–69 years, between 2006 and 2010. Baseline assessments were conducted at 22 research centres located across the United Kingdom [5]. Participants completed touchscreen questionnaires to provide information on sociodemographic factors (including age, gender, ethnicity, and postcode of residence), lifestyle (including smoking status and alcohol intake) and medical history. We excluded participants who reported chronic neurological diseases which could directly affect cognitive function (see S1 Table).

Participants provided full informed consent to participate in UK Biobank. This study was covered by the generic ethical approval for UK Biobank studies from the NHS National Research Ethics Service (approval letter dated 17^th^ June 2011, Ref 11/NW/0382).

### Materials and procedure

#### Baseline cognitive data

Five cognitive tests were included in UK Biobank, all of which were administered via computerised touchscreen interface.

*Numeric memory*: A subset of participants completed a test of numeric memory (‘Maximum digits remembered’; http://biobank.ctsu.ox.ac.uk/crystal/field.cgi?id=4282). Participants were shown a two-digit number which they were asked to recall after a brief pause. This increased by one until the participant made an error, or they reached the maximum of twelve digits.

*Reasoning*: A task with thirteen logic/reasoning-type questions and a two-minute time limit was labelled as ‘fluid intelligence’ in the UK Biobank protocol but is hereafter referred to as ‘verbal-numerical reasoning’; http://biobank.ctsu.ox.ac.uk/crystal/field.cgi?id=20016). The maximum score is 13. The Cronbach alpha coefficient for these items has been reported elsewhere as 0.62 [11].

*Reaction time*: Participants completed a timed test of symbol matching, similar to the common card game ‘Snap’ hereafter referred to as reaction time (RT) (http://biobank.ctsu.ox.ac.uk/crystal/field.cgi?id=20023). The score on this task was the mean response time in milliseconds across trials which contained matching pairs. Cronbach’s alpha for this task has previously been reported as 0.85 [11].

*Visual memory*: A visual memory test was administered, labelled ‘pairs-matching’ (http://biobank.ctsu.ox.ac.uk/crystal/label.cgi?id=100030), where participants were asked to memorize the positions of six card pairs, and then match them from memory while making as few errors as possible. Scores on the pairs-matching test are for the number of errors that each participant made; therefore, higher scores reflect poorer cognitive function. The Pairs matching task had two versions: 3-pair and 6-pair. We focussed analysis on the 6-pair version because there was greater scope for score variation. We refer to this test as the visual memory task from here on.

*Prospective Memory*: For the Prospective Memory (PM) test, participants were asked to engage in a specific behaviour later in the assessment: ‘At the end of the games we will show you four coloured symbols and ask you to touch the blue square. However, to test your memory, we want you to actually touch the Orange Circle instead’ (‘Prospective memory result; http://biobank.ctsu.ox.ac.uk/crystal/field.cgi?id=20018). We scored participants as zero or one, depending on whether they completed the task on first attempt or not.

The reasoning and PM tasks were only added to the participant assessment part-way through the baseline assessment phase. The numeric memory task was added and subsequently removed due to time constraints. Therefore, the sample sizes for the different tasks vary. The cognitive data in UK Biobank have been described previously [12].

#### Demographic data

Education was based on self-report of the highest qualification achieved and dichotomised into university/college degree or less. Participants self-reported their ethnic group and we recoded this into white and non-white [13]. Detailed lifestyle factors such as alcohol intake have been reported elsewhere, stratified by sex and ethnicity[14].

#### Repeat cognitive data

Of the baseline 502,649 UK Biobank participants, a sub-sample of 20,339 underwent repeat assessment between August 2012 and June 2013 and 20,346 provided sufficient data (e.g. age at baseline and re-test). The interval between assessment dates varied between participants (mean = 4.33 years, SD = 0.93, range = 2 to 7). Participants who took part in the repeated assessment all lived with 35 kilometres of the Stockport (UK) Biobank coordinating centre, with a 21% response rate to the invitation email/letter. All participants underwent repeat physical, medical, sociodemographic and cognitive assessment. Because some cognitive tasks were added/removed at different stages of baseline assessment, the number of participants with complete two-wave data varies across tests. Note that the numeric memory task was not included in the repeat assessment battery, and so only four tests–reasoning, RT, visual memory and PM, are considered.

### Analysis

Because the RT scores were significantly positively distributed we transformed the variable with a natural log transform (‘LN’). The visual memory error scores were significantly skewed and zero-value inflated and therefore transformed with an LN +1 equation, both in IBM SPSS V.22. For log RT and log visual memory scores we report exponentiated betas; the effects are multiplicative so that an effect size of 1.00 represents no change, and e.g. beta = 1.01 equates to a 1% increase in the raw scores. For reasoning and numeric memory scores we report unstandardized betas. Because of the large sample sizes, we set our threshold of statistical significance at P = 0.01, partly to offset spurious findings through multiple comparisons.

Data reduction was applied to the baseline assessment cognitive test scores, using PCA. We included four of the cognitive variables assessed in UK Biobank: log RT, verbal-numerical reasoning, numeric memory, and log visual memory errors. We did not include PM in the PCA of cognitive ability because it has a binary outcome and therefore limited variance. Strictly speaking, PCA does not produce latent ‘factors’ however this usage is common and adopted here [15]. Because the numeric memory N was much lower than other tests we ran PCA three times to compare the outputs: at first with all four tests and then with numeric memory removed, and then log visual memory errors removed, leaving just reasoning and log RT. Inter-correlations between cognitive tests were examined with Pearson bivariate correlations, except for analyses including PM (a binary variable) where we instead report point-biserial *r* coefficients.

For the longitudinal cognitive data, we analysed stability in cognitive scores in two ways: relative consistency (i.e. rank consistency; higher scores at baseline predict higher at follow-up), and absolute consistency (i.e. similar raw scores [16]). To examine relative consistency, we ran 2,1 (‘two-way random’) intraclass correlations (ICC), often simplified to ICC (2,1 [17]) which are designed to account for systematic error (e.g. prevalent practice effects) and also random error in cognitive scores (e.g. individual alertness[18]). In terms of absolute reliability, we ran unadjusted repeated-measures ANOVA to test for significant differences in scores. From the ANOVA statistics we also calculated the ‘smallest real difference’ (SRD) value, an index that can be used to define the difference between two scores needed to reflect ‘real’ differences, excluding systematic and random error [16]. The UK Biobank cohort will be followed up and re-tested on the described cognitive measures, and future researchers investigating cognitive decline may find it helpful to know the magnitude of change in a given individual that is likely to represent true change rather than possible measurement imprecision. The SRD is also known as the repeatability coefficient [19]. This was calculated using the formula: “standard error of measurement (SEM) multiplied by 1.96 multiplied by √2” (where 1.96 reflects the Z score associated with a 95% CI [16]). Any change in a participant’s score that is greater than the SRD is considered a ‘real’ change. Note that we did not adjust for age or interval in the SRD score calculation, because the aim here was to quantify the amount of raw change rather than to account for it. We also re-ran the SRD analyses in participants over the age of 60 at baseline, where we may expect greater cognitive decline due to older age [20].

To test for an effect of test-retest interval length, and a potential moderating/interactive role of baseline age (e.g. where participants at older ages may show greater relative change in scores), we also ran repeated-measures ANOVA’s that included baseline age and interval in years (between test sessions) in the model as well as two- and three-way interaction terms including time point, baseline age, and length of interval.

Because the PM task is a binary variable, we computed absolute agreement (in percentage); we formally tested for PM score agreement with Cohen’s kappa, which measures ‘true’ agreement beyond chance, although this can be very conservative.

## Results

Detailed descriptive statistics are shown in Table 1. Of 502,649 participants we excluded 22,221 (4.4%) with a neurological disease (S1 Table). We also excluded 12 individuals who were not within the formal UK Biobank age limit of 40–70, because they inflated the age/cognitive score error bars. This left a total of 480,416 participants.

**Table 1:** Descriptive statistics for the cognitive and demographic data in UK Biobank.

*Baseline cognitive tests*	*N*	Mean (SD) if applicable
Numeric memory score	48,335	6.70 (1.34)
Verbal-numerical reasoning score	158,845	5.99 (2.16)
Log Reaction time	475,051	5.63 (0.19)
Reaction time in milliseconds, median (IQR)	475,051	546 (130)
Log visual memory errors	475,948	1.44 (0.66)
Raw visual memory errors, median (IQR)	475,948	3 (3)
Prospective memory; successful N (%)	134,201	126,007 (93.9)
*Demographic/lifestyle variables*		
Age in years	480,416	56.44 (8.10)
Gender, male N (%)	480,416	217,723 (45.3)
Degree obtained, yes (%)	475,235	155,238 (32.7)
Ethnicity, white (%)	477,772	451,528 (94.5)

*Note*: IQR = interquartile range; SD = standard deviation. White ethnicity refers to participants that reported themselves at assessment as ‘White’, ‘White; British’, ‘White; Irish’, or ‘Any other White background’.

### Data reduction and cognitive score inter-correlations

Unadjusted correlations showed that the tests associated significantly and in the expected directions (e.g. faster RT and higher Reasoning scores; Table 2). All correlations were of small-to-medium effect size; the strongest correlation (in terms of *r*) was between reasoning and numeric memory scores (*r* = 0.391, P < 0.001), and the weakest was between memory errors and log RT (*r* = 0.123, P <0.001). The PM scores showed generally weak correlations; the strongest was with reasoning scores (*r* = 0.250, P <0.001).

**Table 2:** Unadjusted inter-correlations between cognitive variables.

	Numeric memory	Verbal-numerical reasoning	Log reaction time	Log visual memory errors	Prospective memory
Numeric memory	-				
Verbal-numerical reasoning	0.391	-			
Log reaction time	-0.130	-0.180	-		
Log visual memory errors	-0.124	-0.190	0.123	-	
Prospective memory	0.188	0.250	-0.133	-0.105	-

Notes: PM-other variable correlations are point-biserial *r* statistics; all other correlations are Pearson *r* coefficients.

**Fig 1:**
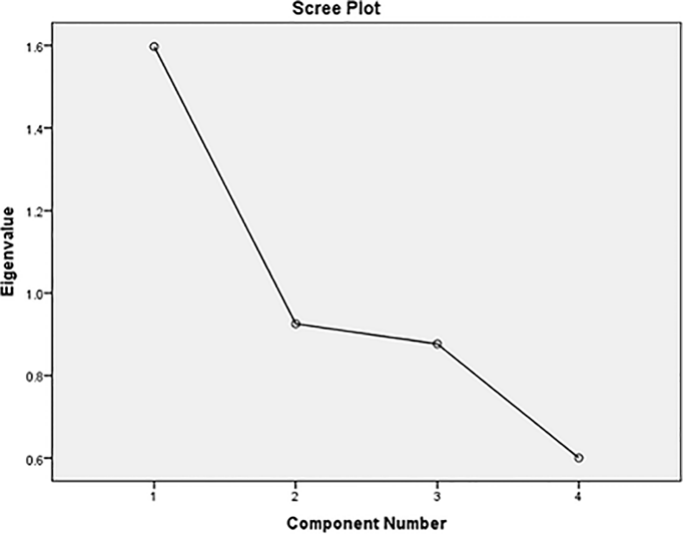
Scree plot for cognitive general factor scores.

The first PCA model included RT, verbal-numerical reasoning, numeric memory and visual memory errors, and had an eigenvalue of 1.60, accounting for 39.93% of the variance. The second model (not including numeric memory) had an eigenvalue of 1.33, accounting for 44.35% of the variance. The third model, not including numeric memory or log visual memory errors, had an eigenvalue of 1.18 and accounted for 59.00% of the variance. All individual test scores had moderate-to-high loadings on their respective first unrotated principal components (range = [–] 0.49 to 0.77; see Table 3). Inspection of eigenvalues and scree-plots suggested that a single component should be extracted from the data in all cases (see Fig 1, which shows the first PCA).

**Table 3:** Factor loadings for principal components analysis of cognitive tests.

	Numeric memory	Verbal-numerical reasoning	Log reaction time	Log visual memory errors
Factor loadings: Model 1	0.716	0.774	-0.491	-0.495
Factor loadings: Model 2	N/A	-0.716	0.632	0.647
Factor loadings: Model 3	N/A	0.768	-0.768	N/A

Note: Scores reflect component loadings.

### Cognitive test score within-participant stability

A subsample of the main UK Biobank sample had complete two-wave data for verbal-numerical reasoning (N = 4,523), RT (N = 19,327), visual memory (N = 19,017) and PM (N = 3,879). Two-way random ICC statistics showed statistically significant correlations for verbal-numerical reasoning (ICC = 0.65, 95% CI = 0.63 to 0.67), log RT (ICC = 0.57; 95% CI = 0.56 to 0.58) and log visual memory errors (ICC = 0.16; 95% CI = 0.15 to 0.17; all P values < 0.001). In order to explore the relatively lower reliability of the visual memory task further, we obtained additional data from the pilot phase of the UK Biobank baseline wave. In this phase, N = 3,598 participants (excluding those with neurological conditions) performed the visual memory task (6-pair version) twice in immediate succession, with ICC for log visual memory errors being 0.17 (95% CI = 0.14 to 0.20; P < 0.001). In those participants who had complete data for the pilot trials and the longitudinal follow-up (N = 447; mean interval = 6.4 years, SD = 0.14, range 6.2 to 7.0), the log visual memory errors ICC between the second pilot trial and the follow-up assessment was 0.13 (95% CI = 0.03, 0.21; P = 0.016).

Cohen’s kappa for PM indicated significant agreement across assessment waves (κ = 0.36, P < 0.001); 3,799 (97.9%) of the 3,879 with data were successful at both baseline and retest sessions.

Repeated measures ANOVA’s showed significant differences across waves 1 and 2 for log RT (F = 110.06, P <0.001) and visual memory errors (F = 42.76, P <0.001; see Table 4). There was a nominal (i.e. significant at the conventional P<0.05) significant difference in verbal-numerical reasoning scores (F = 5.32, P = 0.021), although not in the context of our more conservative alpha of 0.01. Scores on verbal-numerical reasoning and visual memory errors improved, while RT scores got worse. In none of these measures were the mean changes in the sample above the estimated ‘Smallest real difference’ (Table 4). We also ran these analyses after stratifying participants into those aged under 60 (S2 Table), and 60 and above (S3 Table). This made no difference to the results.

**Table 4:** Cognitive test scores at Time 1 and Time 2, and reliability statistics.

		Time 1	Time 2	Relative reliability indices				Absolute reliability indices			
	N	Mean (SD)	Mean (SD)	*r*	ICC (2,1)	P-value	F-value	P-value	Mean square residual	SEM	Smallest real difference
Verbal-numerical reasoning	4,523	6.95 (2.01)	7.00 (2.01)	0.65	0.65	<0.001	5.32	0.021	1.42	1.19	3.30
Log reaction time	19,327	6.29 (0.17)	6.30 (0.18)	0.57	0.57	<0.001	110.06	<0.001	0.01	0.12	0.28
(untransformed)		548.36 (102.67)	556.09 (109.34)	0.54	0.54	<0.001	111.79	<0.001	5162.00	71.85	199.15
Log visual memory errors	19,017	1.42 (0.64)	1.38 (0.63)	0.16	0.16	<0.001	42.76	<0.001	0.34	0.58	1.61
(untransformed)		5.00 (3.17)	4.80 (3.05)	0.19	0.19	<0.001	51.80	<0.001	7.82	2.80	7.75

Notes: SD = standard deviation. ICC = intraclass correlation. SEM = standard error of measurement, equivalent to the square root of mean square residual[16]. Smallest real difference = ‘SEM * 1.96 * √2’. F-value = within participants ANOVA.

We tested for interactions between time point (i.e. first or second assessment), baseline age, and interval (in days between sessions; all two- and three-way). Of these, there were no interactions significant at P <0.01.

## Discussion

### Overview

Aspects of the cognitive assessment used in UK Biobank were novel because of the need to conduct brief computerized cognitive assessments in a very large population. This paper demonstrates two points. The first point is that the UK Biobank cognitive data significantly inter-correlates and can be used to make a ‘*g*-factor’ score of general cognitive ability, however the amount of variance explained by that factor score is not as high as in other cohorts [15,21]. The second is that the cognitive scores in participants with repeat testing, on average four years later, showed reasonable stability and principally showed test-retest reliability for the reasoning and RT tasks. The visual memory task did not show good stability across time. The UK Biobank cognitive data promises to be a valuable resource for future studies.

### Interpretation

In terms of factor analysis with PCA, eigenvalues and scree-plots indicated an unrotated one-factor solution for the cognitive data. It is important to describe the *g*-factor score because it may be particularly valuable to investigations of general cognitive ability, e.g. genetic studies. The factor solution based on four measures accounted for around 40% of total variance, slightly less than in cohorts with more detailed assessments which report 40–50% [21].

Longitudinal cognitive data showed that participants on average improved very slightly on the reasoning and visual memory tasks. These improvements may reflect practice effects [22]. Aspects of the reasoning assessment may measure a degree of ‘crystallised-type’ knowledge (e.g. ‘synonyms’); this type of ability would not be expected to deteriorate with age. Note that the magnitude of cognitive change was very small for all tests. Intraclass correlations showed that reasoning and RT tasks showed good stability across time points. However, while statistically significant, the estimate was low for the visual memory task (under 0.20); this was the case when this test was repeated either immediately or after several years, and regardless of whether participants performed it twice or three times. For comparison, Johnson et al. reported a raw Pearson *r* correlation around 0.70 for each of several commonly used memory tests (Logical Memory total, Verbal Paired Associates and Digit Span Backwards/Letter-number sequencing tests), between the ages of 70 and 73 in the Lothian Birth Cohort 1936 [23]. The ‘Smallest real difference’ values suggested that relatively large differences in scores would be required in order to show meaningful change across time points, potentially suggesting a degree of measurement imprecision in the tests. Research based on the baseline cognitive data available in UK Biobank (i.e. collected between 2006 and 2010), should take these limitations into account.

### Limitations and future research

It is beneficial to have participants assessed on several tests of each cognitive domain, allowing for general factors of (for example) processing speed or memory, because these reflect only the shared variance between tests and therefore less task-specific variance[9]. Because the assessment did not include two or more cognitive tests that ostensibly test the same domain, it is not possible to create cognitive domain scores that are intermediate between the specific test and *g* (e.g. ‘memory’ [24,25]). More detailed cognitive assessment will be included in future UK Biobank waves, allowing researchers to say with greater certainty that each cognitive test assesses what it intends to (in terms of cognitive domains).

It is possible that findings are affected by a degree of ‘practice effects’ in the repeat-assessment sample, where the change in cognitive test scores that may be expected across time is influenced (to some degree) by familiarity with the tests [24]. Previous studies indicate small-to-null individual differences in the impact of cognitive practice effects [22]. It is possible with these novel UK Biobank-specific tasks, however, that some aspects of the battery are more susceptible to practice effects than others.

It is also possible that a degree of floor/ceiling effects are prevalent in some of the tests (i.e. where a task’s characteristics give it an upper or lower limit where it cannot distinguish between true differences in participant abilities [26]). For example, a large number of participants scored zero or very few errors on the visual memory task, and the PM score was analysed as a binary variable; these factors may contribute to the relatively low reliability shown by these tests. There may be a degree of selection bias in terms of less impaired individuals attending baseline and then repeat assessment.

### Summary

This report characterizes and describes the cognitive data in UK Biobank in important ways, demonstrating a valid *g* factor of mental ability, and showing general stability in cognitive scores in a sub-sample of participants with longitudinal data. The UK Biobank promises to be a valuable resource for researchers seeking determinants of cognitive ability, and related outcomes. Future research will examine potential moderators of the cognitive test scores reported here.

## Supporting Information

S1 Table Excluded (self-reported) diseases. (DOCX)

S2 Table Cognitive test scores at Time 1 and Time 2, and reliability statistics in participants aged 40 to 59. (DOCX)

S3 Table Cognitive test scores at Time 1 and Time 2, and reliability statistics in participants aged 60 and over. (DOCX)

## References

[1] Batty GD, Deary IJ, Gottfredson LS. Premorbid (early life) IQ and Later Mortality Risk: Systematic Review. Annals of Epidemiology. 2007 pp. 278–288. 10.1016/j.annepidem.2006.07.010 17174570

[2] Calvin CM, Deary IJ, Fenton C, Roberts BA, Der G, Leckenby N, et al Intelligence in youth and all-cause-mortality: Systematic review with meta-analysis. Int J Epidemiol. 2011;40: 626–644. 10.1093/ije/dyq190 21037248 PMC3147066

[3] Martin GM. Defeating dementia. Nature. 2004 pp. 247–248. 10.1038/431247b

[4] Prince M, Bryce R, Albanese E, Wimo A, Ribeiro W, Ferri CP. The global prevalence of dementia: a systematic review and metaanalysis. Alzheimers Dement. 2013;9: 63–75.e2. 10.1016/j.jalz.2012.11.007 23305823

[5] Allen N, Sudlow C, Downey P, Peakman T, Danesh J, Elliott P, et al UK Biobank: Current status and what it means for epidemiology. Heal Policy Technol. 2012;1: 123–126. 10.1016/j.hlpt.2012.07.003

[6] Sudlow C, Gallacher J, Allen N, Beral V, Burton P, Danesh J, et al UK Biobank: An Open Access Resource for Identifying the Causes of a Wide Range of Complex Diseases of Middle and Old Age. PLoS Med. Public Library of Science; 2015;12: e1001779 10.1371/journal.pmed.1001779 25826379 PMC4380465

[7] Deary IJ, Penke L, Johnson W. The neuroscience of human intelligence differences. Nat Rev Neurosci. Nature Publishing Group; 2010;11: 201–11. 10.1038/nrn2793 20145623

[8] Smith DJ, Nicholl BI, Cullen B, Martin D, Ul-Haq Z, Evans J, et al Prevalence and characteristics of probable major depression and bipolar disorder within UK Biobank: Cross-sectional study of 172,751 participants. PLoS One. 2013;8 10.1371/journal.pone.0075362 PMC3839907 24282498

[9] Burns RB, Carroll JB. Human Cognitive Abilities: A Survey of Factor-Analytic Studies. Educational Researcher 1994 p. 35 10.2307/1177226

[10] Trampush JW, Lencz T, Knowles E, Davies G, Guha S, Pe’er I, et al Independent evidence for an association between general cognitive ability and a genetic locus for educational attainment. Am J Med Genet B Neuropsychiatr Genet. 2015; 10.1002/ajmg.b.32319 PMC4500051 25951819

[11] Hagenaars SP, Harris SE, Davies G, Hill WD, Liewald DCM, Ritchie SJ, et al Shared genetic aetiology between cognitive functions and physical and mental health in UK Biobank (N = 112151) and 24 GWAS consortia. Mol Psychiatry. Macmillan Publishers Limited; 2016; 10.1038/mp.2015.225 PMC5078856 26809841

[12] Cullen B, Nicholl BI, Mackay DF, Martin D, Ul-Haq Z, McIntosh A, et al Cognitive function and lifetime features of depression and bipolar disorder in a large population sample: Cross-sectional study of 143,828 UK Biobank participants. Eur Psychiatry. 2015;30: 950–958. 10.1016/j.eurpsy.2015.08.006 26647871

[13] Tyrrell JS, Yaghootkar H, Freathy RM, Hattersley AT, Frayling TM. Parental diabetes and birthweight in 236 030 individuals in the UK Biobank study. Int J Epidemiol. 2013;42: 1714–1723. 10.1093/ije/dyt220 24336895 PMC3887570

[14] Ntuk U, Gill J, Mackay D, Sattar N, Pell J. Ethnic specific obesity cut-offs for diabetes risk: cross-sectional study of 490, 288 UK Biobank participants. Diabetes Care. American Diabetes Association; 2014 10.2337/dc13-2966 < 10.2337/dc13-2966>) 24974975

[15] Houlihan LM, Wyatt ND, Harris SE, Hayward C, Gow AJ, Marioni RE, et al Variation in the uric acid transporter gene (SLC2A9) and memory performance. Hum Mol Genet. 2010;19: 2321–2330. 10.1093/hmg/ddq097 20197412

[16] Weir JPJ. Quantifying test-retest reliability using the intraclass correlation coefficient and the SEM. J Strength Cond Res. 2005;19: 231–40. 10.1519/15184.1 15705040

[17] Shrout PE, Fleiss JL. Intraclass correlations: uses in assessing rater reliability. Psychol Bull. 1979;86: 420–8. Available: http://www.ncbi.nlm.nih.gov/pubmed/18839484 18839484 10.1037//0033-2909.86.2.420

[18] Vaz S, Falkmer T, Passmore AE, Parsons R, Andreou P. The case for using the repeatability coefficient when calculating test-retest reliability. PLoS One. Public Library of Science; 2013;8: e73990 10.1371/journal.pone.0073990 24040139 PMC3767825

[19] Bland JM, Altman DG. Statistics Notes: Measurement error. BMJ. 1996;313: 744–744. 10.1136/bmj.313.7059.744 8819450 PMC2352101

[20] Marioni RE, Campbell A, Scotland G, Hayward C, Porteous DJ, Deary IJ. Differential effects of the APOE e4 allele on different domains of cognitive ability across the life-course. Eur J Hum Genet. Macmillan Publishers Limited; 2015; 10.1038/ejhg.2015.210 PMC4705436 26395552

[21] Lyall DM, Lopez LM, Bastin ME, Maniega SM, Penke L, Valdés Hernández M del C, et al ADRB2, brain white matter integrity and cognitive ageing in the Lothian Birth Cohort 1936. Behav Genet. 2013;43: 13–23. 10.1007/s10519-012-9570-x 23229623

[22] Salthouse TA, Tucker-Drob EM. Implications of short-term retest effects for the interpretation of longitudinal change. Neuropsychology. 2008 10.1037/a0013091 PMC2593909 18999354

[23] Johnson W, Gow A, Corley J, Redmond P, Henderson R, Murray C, et al Can we spot deleterious ageing in two waves of data? The Lothian Birth Cohort 1936 from ages 70 to 73. Longit Life Course Stud. 2012;3: 312–331. 10.14301/llcs.v3i3.198

[24] Salthouse TA. Why Are There Different Age Relations in Cross-Sectional and Longitudinal Comparisons of Cognitive Functioning? Curr Dir Psychol Sci. 2014;23: 252–256. 10.1177/0963721414535212 25382943 PMC4219741

[25] Deary IJ, Corley J, Gow AJ, Harris SE, Houlihan LM, Marioni RE, et al Age-associated cognitive decline. British Medical Bulletin. 2009 pp. 135–152. 10.1093/bmb/ldp033 19776035

[26] Sternberg RJ. Handbook of Intelligence. Cambridge University Press; 2000 Available: https://books.google.com/books?hl=en&lr=&id=YnBGMpIMfJ0C&pgis=1

