# Correction: Cognitive Test Scores in UK Biobank: Data Reduction in 480,416 Participants and Longitudinal Stability in 20,346 Participants

**DOI:** 10.1371/journal.pone.0156366

**Published:** 2016-05-20

**Authors:** Donald M. Lyall, Breda Cullen, Mike Allerhand, Daniel J. Smith, Daniel Mackay, Jonathan Evans, Jana Anderson, Chloe Fawns-Ritchie, Andrew M. McIntosh, Ian J. Deary, Jill P. Pell

The affiliation for the eighth author is incorrect. Chloe Fawns-Ritchie is not affiliated with #1 but with #2: Centre for Cognitive Ageing and Cognitive Epidemiology, Department of Psychology, University of Edinburgh, Edinburgh, United Kingdom.
