# Supplementary material for: Cognitive Test Scores in UK Biobank: Data Reduction in 480,416 Participants and Longitudinal Stability in 20,346 Participants
Source: PLoS One. 2016 Apr 25;11(4):e0154222. doi: 10.1371/journal.pone.0154222 (PMC4844168; doi:10.1371/journal.pone.0154222)
Supplement: S1 Table — (DOCX) [file pone.0154222.s001.docx]

**S1 Table.** Excluded (self-reported) diseases.

Brain cancer/primary malignant tumour

Brain haemorrhage

Brain/intracranial abscess

Cerebral aneurysm

Cerebral palsy

Chronic/degenerative neurological problem

Dementia/Alzheimer's disease/cognitive impairment

Encephalitis

Epilepsy

Head injury

Infection of nervous system

Ischaemic stroke

Meningeal cancer/malignant meningioma

Meningioma (benign)

Meningitis

Motor neurone disease

Multiple sclerosis

Neurological injury/trauma

Neuroma (benign)

Other demyelinating condition

Other neurological problem

Parkinson's disease

Spina bifida

Stroke

Subarachnoid haemorrhage

Subdural haematoma

Transient ischaemic attack
