# Supplementary material for: Cognitive Test Scores in UK Biobank: Data Reduction in 480,416 Participants and Longitudinal Stability in 20,346 Participants
Source: PLoS One. 2016 Apr 25;11(4):e0154222. doi: 10.1371/journal.pone.0154222 (PMC4844168; doi:10.1371/journal.pone.0154222)
Supplement: S2 Table — (DOCX) [file pone.0154222.s002.docx]

**S2 Table.** Cognitive test scores at Time 1 and Time 2, and reliability statistics in participants aged 40 to 59.

|  |  | Time 1 | Time 2 | Relative reliability indices | | | | | Absolute reliability indices | | |
| --- | --- | --- | --- | --- | --- | --- | --- | --- | --- | --- | --- |
|  | N | Mean (SD) | Mean (SD) | *r* | ICC (2,1) | P-value | F-value | P-value | Mean square residual | SEM | Smallest real difference |
| Verbal-numerical reasoning | 2,316 | 7.06 (2.04) | 7.15 (2.03) | 0.66 | 0.66 | <0.001 | 6.76 | 0.009 | 1.42 | 1.19 | 3.31 |
| Log reaction time | 10,777 | 6.26 (0.17) | 6.26 (0.17) | 0.56 | 0.56 | <0.001 | 13.16 | <0.001 | 0.01 | 0.11 | 0.32 |
| (untransformed) |  | 529.76 (96.57) | 533.24 (100.57) | 0.53 | 0.53 | 0<0.001 | 14.26 | <0.001 | 4751.52 | 68.93 | 191.07 |
| Log visual memory errors | 10,538 | 1.37 (0.63) | 1.32 (0.62) | 0.16 | 0.16 | <0.001 | 31.92 | <0.001 | 0.33 | 0.57 | 1.59 |
| (untransformed) |  | 4.73 (2.99) | 4.49 (2.84) | 0.19 | 0.19 | <0.001 | 41.41 | <0.001 | 6.90 | 2.63 | 7.28 |

Notes: SD = standard deviation. ICC = intraclass correlation. SEM = standard error of measurement, equivalent to the square root of mean square residual[16]. Smallest real difference = ‘SEM * 1.96 * $\surd$2’. F-value = within participants ANOVA.
