# Supplementary material for: Cognitive Test Scores in UK Biobank: Data Reduction in 480,416 Participants and Longitudinal Stability in 20,346 Participants
Source: PLoS One. 2016 Apr 25;11(4):e0154222. doi: 10.1371/journal.pone.0154222 (PMC4844168; doi:10.1371/journal.pone.0154222)
Supplement: S3 Table — (DOCX) [file pone.0154222.s003.docx]

**S3 Table.** Cognitive test scores at Time 1 and Time 2, and reliability statistics in participants aged 60 and over.

|  |  | Time 1 | Time 2 | Relative reliability indices | | | | | Absolute reliability indices | | |
| --- | --- | --- | --- | --- | --- | --- | --- | --- | --- | --- | --- |
|  | N | Mean (SD) | Mean (SD) | *r* | ICC (2,1) | P-value | F-value | P-value | Mean square residual | SEM | Smallest real difference |
| Verbal-numerical reasoning | 2,207 | 6.82 (1.96) | 6.84 (1.97) | 0.64 | 0.64 | <0.001 | 0.40 | 0.526 | 1.41 | 1.19 | 3.29 |
| Log reaction time | 8,550 | 6.33 (0.17) | 6.35 (0.18) | 0.53 | 0.53 | <0.001 | 131.24 | <0.001 | 0.02 | 0.12 | 0.34 |
| (untransformed) |  | 571.82 (105.30) | 584.90 (113.10) | 0.51 | 0.50 | <0.001 | 124.440 | <0.001 | 5881.16 | 76.69 | 212.57 |
| Log visual memory errors | 8,479 | 1.49 (0.64) | 1.46 (0.63) | 0.14 | 0.14 | <0.001 | 12.39 | <0.001 | 0.35 | 0.59 | 1.63 |
| (untransformed) |  | 5.35 (3.34) | 5.18 (3.25) | 0.18 | 0.18 | <0.001 | 14.244 | <0.001 | 8.97 | 2.99 | 8.30 |

Notes: SD = standard deviation. ICC = intraclass correlation. SEM = standard error of measurement, equivalent to the square root of mean square residual[16]. Smallest real difference = ‘SEM * 1.96 * $\surd$2’. F-value = within participants ANOVA.
